# Utility of DNA barcoding to identify rare endemic vascular plant species in Trinidad

**DOI:** 10.1002/ece3.3220

**Published:** 2017-08-09

**Authors:** Fazeeda N. Hosein, Nigel Austin, Shobha Maharaj, Winston Johnson, Luke Rostant, Amanda C. Ramdass, Sephra N. Rampersad

**Affiliations:** ^1^ Faculty of Science and Technology Department of Life Sciences The University of the West Indies St. Augustine Trinidad and Tobago – West Indies

**Keywords:** biodiversity conservation, DNA barcoding, endemics, molecular genetics

## Abstract

The islands of the Caribbean are considered to be a “biodiversity hotspot.” Collectively, a high level of endemism for several plant groups has been reported for this region. Biodiversity conservation should, in part, be informed by taxonomy, population status, and distribution of flora. One taxonomic impediment to species inventory and management is correct identification as conventional morphology‐based assessment is subject to several caveats. DNA barcoding can be a useful tool to quickly and accurately identify species and has the potential to prompt the discovery of new species. In this study, the ability of DNA barcoding to confirm the identities of 14 endangered endemic vascular plant species in Trinidad was assessed using three DNA barcodes (*matK*,* rbcL*, and *rpoC1*). Herbarium identifications were previously made for all species under study. *matK*,* rbcL,* and *rpoC1* markers were successful in amplifying target regions for seven of the 14 species. *rpoC1* sequences required extensive editing and were unusable. *rbcL* primers resulted in cleanest reads, however, *matK* appeared to be superior to *rbcL* based on a number of parameters assessed including level of DNA polymorphism in the sequences, genetic distance, reference library coverage based on BLASTN statistics, direct sequence comparisons within “best match” and “best close match” criteria, and finally, degree of clustering with moderate to strong bootstrap support (>60%) in neighbor‐joining tree‐based comparisons. The performance of both markers seemed to be species‐specific based on the parameters examined. Overall, the Trinidad sequences were accurately identified to the genus level for all endemic plant species successfully amplified and sequenced using both *matK* and *rbcL* markers. DNA barcoding can contribute to taxonomic and biodiversity research and will complement efforts to select taxa for various molecular ecology and population genetics studies.

## 1 INTRODUCTION

Trinidad and Tobago (latitudes 10.0°N and 11.3°N and longitudes 60.3°W and 62°W) are the southernmost islands in the Caribbean and are bordered by the Atlantic Ocean and Caribbean Sea. The islands are located 11.3 and 32 km northeast of the Venezuelan coast of South America (Lesser Antilles; Kenny, Comeau, & Katwaru, 1997; Kenny, 2008). Although both islands are positioned on the South American Continental Shelf, it was proposed that Trinidad separated from the South American continent later (ca. 1,500 years ago) than Tobago (ca. 11,000–13,000 years ago; Kenny, 2008). The flora of Trinidad and Tobago is estimated to include 2, 407 vascular plant species of which approximately 4.5% are endemic (Baksh‐Comeau et al., 2016). This level of endemism is reflective of the close proximity to, and the relatively short geological time frame since separation from the South American mainland, and cannot be compared with oceanic islands in the Greater Antilles, such as Jamaica (12%–50% endemism; MacArthur, 1972; van den Eynden, Oatham, & Johnson, 2008; Baksh‐Comeau et al., 2016).

The Caribbean islands are among the world's most important “biodiversity hotspots” (Mittermeier et al., 2004; Shi, Singh, Kant, Zhu, & Waller, 2005), and, in global terms, can be compared to the Madagascar and Cape Floristic hotspots in terms of the number of endemic genera (Francisco‐Ortega et al., 2007; Maunder et al., 2008; Mittermeier et al., 2004). Based on the International Union for Conservation of Nature (IUCN) Red List categories, and the Global Star rating system, species located in hotspots of high conservation value should be inventoried to assess the distribution and population status of endemics (Baksh‐Comeau et al., 2016). The conservation of natural plant resources in the Caribbean is especially critical for providing essential ecosystem services (Kress & Horvitz, 2005). However, in many biodiversity hotspots, the botanical inventory is usually incomplete, perhaps because taxonomic assignment is frustrated by low discriminatory power of morphological descriptors for very closely related species (Francisco‐Ortega et al., 2007; Zanoni, 1989). DNA barcoding has the potential to support species identification and discovery, vegetation, and floristic species surveys, in addition to studies on ecological forensics, all of which are critical to biodiversity management (Hollingsworth, Li, van der Bank, & Twyford, 2016; Valentini, Pompanon, & Taberlet, 2008; von Crautlein, Korpelainen, Pietiläinen, & Rikkinen, 2011).

Apart from biodiversity conservation, accurate taxonomic assignment is important to the practice of traditional or herbal medicine (Techen, Parveen, Pan, & Khan, 2014). In the Caribbean, herbal remedies are referred to as “bush medicine” (Laguerre, 1987; Mahabir & Gulliford, 1997; Quinlan & Quinlan, 2007). In Trinidad, approximately one‐third of the flora is composed of exotic species which are used as bush medicines according to an ethno‐botanical survey conducted between 2007 and 2008 (Clement, Baksh‐Comeau, & Seaforth, 2015). The danger of collecting plants for use as herbal remedies lies in some medicinal plant species having multiple synonyms, in addition to having a vernacular name, which may be mistakenly used to identify more than one plant species (Bellakhdar, Claisse, Fleurentin, & Younos, 1991). Endangered species may be mistakenly collected to extinction if their identity is confused with more abundant morphologically similar‐looking individuals. Consumption of plant material from misidentified species could also result in serious health risks to end users (Barthelson, Sundareshan, Galbraith, & Woosley, 2006; Bruni et al., 2010; Mahabir & Gulliford, 1997). For example, the Food and Drug Association (FDA) has advised that consumption of products containing aristolochic acid (derived from plants belonging to the *Aristolochia* genus) has been associated with permanent kidney damage, and development of certain cancers associated with the urinary tract (http://www.cfsan.fda.gov). Similarly, toxic effects have been reported for fruit and leaf consumption of plant species of the genus *Ilex* (Weiner & Weiner, 1999) and *Maytenus* (Da Silva, Serrano, & Silva, 2011). *Aristolochia*,* Ilex,* and *Maytenus* sp. are used in the Caribbean for their proposed medicinal properties (Mahabir & Gulliford, 1997). Preservation of indigenous knowledge concerning medical ethno‐botany is a key aspect of bioprospecting with the *proviso* of accurate species identification (Harvey & Gericke, 2011; Kumar, Sharma, & Chattopadhyay, 2013; Theodoridis et al., 2012).

Conventionally, taxonomic assignment has been the purview of taxonomic experts (Waugh, 2007), however, DNA barcoding may enable rapid and accurate species identification by nonspecialists using nucleotide comparisons of approved gene regions (Coissac, Hollingsworth, Lavergne, & Taberlet, 2016; Hebert, Cywinska, Ball, & deWaard, 2003). A 648‐basepair region of the mitochondrial *cytochrome c oxidase* 1 gene (“*CO1*”) is the accepted barcode for almost all animal groups but, it is not a useful barcode in plants because this region (i) has a slow rate of evolution (Chase & Fay, 2009), (ii) is prone to structural rearrangements (Kelly, Ameka, & Chase, 2010; Palmer et al., 2000), and (iii) does not accommodate for the existence of interspecific and intergeneric hybrids in plants (Rieseberg, Wood, & Baack, 2006). Selection of a plant DNA barcode must meet a number of criteria which have already been described elsewhere (Ford et al., 2009; Hollingsworth et al., 2009; Kress & Erickson, 2008; Li et al., 2015). The chloroplast *ribulose‐1, 5‐bisphosphate carboxylase/oxygenase* large subunit gene (*rbcL*) and *maturase K* gene (*matK*) are the approved barcodes for land plants (CBOL Plant Working Group 2009). However, plant‐plastid barcodes typically have lower resolving power to separate closely related plant species compared to the animal barcode, and in several cases, conspecifics or recently diverged species do not form highly supported, distinct sequence clusters that allow species discrimination (Hollingsworth et al., 2016; van Velzen, Weitschek, Felici, & Bakker, 2012; Zhang et al., 2012). In fact, a uniquely identified species in a given genus is the exception rather than the rule in most plant barcoding studies (Hollingsworth, Graham, & Little, 2011). For these reasons, standard plant barcodes are more appropriately used as “molecular augmentations” to preexisting herbarium identifications as the current plant barcode sequences do not contain sufficient variation to define a species‐level framework for every plant species (Hollingsworth et al., 2016). Further, in using plant barcodes, it is important to understand the limited resolving power of the technique when formulating the objectives of a particular study (Hollingsworth et al., 2016). There are other practical issues to consider which include but are not limited to, the requirement for species‐specific primer combinations which directly determines recovery of *matK* sequences, DNA extraction methods for recalcitrant species whose genomic DNA may be contaminated with PCR inhibitors, for example, muco‐polysaccharides, proteins, polyphenols, and tannins, the need for and expense involved in automated DNA extraction for high‐throughput processing of large sample sizes, and the difficulty in constructing reference sequence datasets or libraries (Hollingsworth et al., 2011, 2016). The most recent development in plant DNA barcoding is to sequence the complete chloroplast genome which will be used as a “super‐barcode” in order to overcome some of the issues associated with low resolving power of the single or multiple loci barcode approach (Hollingsworth et al., 2016; Li et al., 2015).

In this study, we evaluated the ability of three DNA barcodes (*matK*,* rbcL*, and *rpoC1*) to identify specimens of 14 vascular endemic plant species in Trinidad which are endangered or vulnerable according to The International Union for Conservation of Nature and Natural Resources (IUCN) Red List criteria.

## 2 MATERIALS AND METHODS

### 2.1 Plant collection

**Figure 1:**
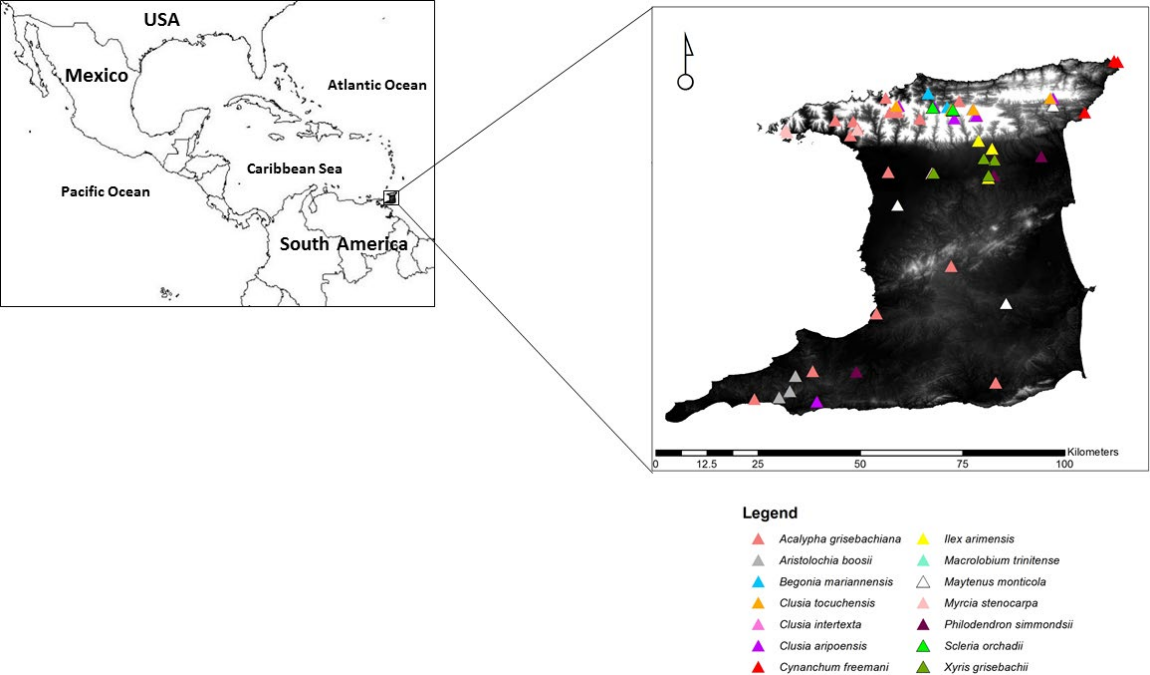
Location map of endemic vascular plant species sampled in this study. The white shaded areas indicate elevation, and it is noted that the majority of endemic species included in this study were located in mountainous regions in North Trinidad

Fourteen endemic vascular plant species were selected for this study (Table 1; Figure 1). Expeditions were led by Mr. Winston Johnson, retired field plant taxonomy expert of the National Herbarium of Trinidad and Tobago. The main consideration for collection was the fact that the majority of species collected were endangered according to the IUCN Red List criteria (Baksh‐Comeau et al., 2016), and this, therefore, restricted the number of individuals collected. In addition, some mountainous species were difficult to retrieve and accessibility was an issue. Five individuals per species per location were collected in labeled bags and transported on ice to the laboratory. Specimen identification and species assignment were independently confirmed prior to DNA analysis and was based on an assessment of morphological descriptors developed by the National Herbarium of Trinidad and Tobago (http://sta.uwi.edu/herbarium/). Voucher specimens were deposited at the National Herbarium of Trinidad and Tobago. Information concerning the endemic species used in this study can be accessed through The National Herbarium of Trinidad and Tobago in conjunction with the University of Oxford through the Darwin Initiative online database, http://herbaria.plants.ox.ac.uk/bol/trin.

**Table 1:** Plant species collection data

Family	Species	IUCN Status^a^
Araceae	*Philodendron simmondsii* Mayo	Endangered
Aristolochiaceae	*Aristolochia boosii* Panter	Endangered
Begoniaceae	*Begonia mariannensis* Wassh. & McClellan	Critically endangered
Caesalpinaceae	*Macrolobium trinitense* Urb.	Near endangered
Celastraceae	*Maytenus monticola* Sandwith	Near threatened
Clusiaceae	*Clusia aripoensis* Britton	Least concern
Clusiaceae	*Clusia intertexta* Britton	Deficient data
Clusiaceae	*Clusia tocuchensis* Britton	Endangered
Cyperaceae	*Scleria orchardii* C.Adams	Vulnerable
Euphorbiaceae	*Acalypha grisebachiana* (Kuntze) Pax & Hoffm.	Vulnerable
Xyridaceae	*Xyris grisebachii* Malme	Critically endangered
Asclepiadaceae	*Cynanchum freemani* (N.E.Br.) Woodson (syn. *Metastelma freemani* N.E. Br.)	Endangered
Aquifoliaceae	*Ilex arimensis* (Loes.) Britton	Least concern
Myrtaceae	*Myrcia stenocarpa* Krug & Urban, Bot. Jahrb. Syst	Near endangered

a IUCN Status—International Union for Conservation of Nature (IUCN) Red List categories.

### 2.2 DNA extraction, amplification, and sequencing

Total genomic DNA was extracted from freshly collected leaf material according to the modified CTAB protocol of Koboyashi, Horikoshi, Katsuyama, Handa, and Takayanagi (1998). The Kobayashi protocol was selected because it is known to successfully extract amplifiable genomic DNA from a number of woody plant tissue with high amounts of polysaccharides including muco‐polysaccharides, polyphenols, and various secondary metabolites such as alkaloids, flavonoids, and phenols, all of which inhibit PCR amplification (Kobayashi et al. 1998). In some cases, the DNA pellet had to be washed up to three times with Buffer 1 and the chloroform‐isoamyl alcohol extraction step was repeated if the aqueous layer was not clear and/or the color of the pellet was brown and sticky according to the recommendations of Koboyashi et al. (1998). DNA extracts were diluted to 10 ng/μl and this served as the initial working DNA concentration for PCR amplification.

Three markers were assessed for species identification: the recommended two‐locus cpDNA barcode (*matK* + *rbcL*; CBOL Plant Working Group 2009), and one cpDNA regions (*rpoC1*; Chase et al., 2005; Kress, Wurdack, Zimmer, Weigt, & Janzen, 2005; Table 2). PCR amplification reagents and thermal conditions were used according to the CBOL laboratory manual guidelines (http://www.barcoding.si.edu/ plant_working_group.html). However, optimization was required for each primer pair and for each species which included the following: (i) varying the concentrations of DMSO, Tween 20, and BSA as PCR enhancers, (ii) titrating the concentration of MgSO_4_, and (iii) assessment of primer annealing temperature through gradient annealing temperature analysis. Optimal amplification was achieved using 1.5 mmol/L MgSO_4_, 1% Tween‐20, 0.8 mg/ml BSA, and 60°C annealing temperature.

**Table 2:** Primer data

Marker/Barcode	Primers F/R	Primer sequence (5′‐3′)	Average amplicon size/bp (amplicon size range)
*rpoC1*	2F	GGCAAAGAGGGAAGATTTCG	494 (462–556)
	4R	CCATAAGCATATCTTGAGTTGG	
*matK*	3F	CGTACAGTACTTTTGTGTTTACGAG	794 (656–861)
	1R	ACCCAGTCCATCTGGAAATCTTGGTTC	
*rbcL*	rbcLa_R	GTAAAATCAAGTCCACCRCG	704 (702–883)
	rbcLa_F	ATGTCACCACAAACAGAGACTAAAGC	

Amplicons were sequenced in both directions by Amplicon Express (Pullman, WA, USA) using Sanger dideoxy sequencing. Verified base calls were carried out by the sequencing company independently as a first check that the sequence reads were correct. The sequences were also checked against the chromatograms using Sequencher v 5.4.1 (https://www.genecodes.com/, Gene Codes Corp., Ann Arbor, Michigan, USA). The data from the *rpoC1* marker was eliminated from further analysis as the sequences were not clean reads despite repeated sequencing attempts. Sequences obtained from the two barcodes were deposited in GenBank (GenBank Accession Nos. KX228511 to KX228515 and KX212893 to KX212899).

### 2.3 Data analysis

Data were analyzed using a compendium of supporting methods as there is no one singular approach that best determines barcoding success in species discrimination (Gong, Liu, Chen, Hong, & Kong, 2016; Mao, Zhang, Nakamura, Guan, & Qiu, 2014). As far as possible, the recommendations for data analysis were followed as outlined by Casiraghi, Labra, Ferri, Galimberti, and De Mattia (2010) and Collins and Cruickshank (2013).

### 2.4 BLAST and reference datasets

Verified representative sequences of each taxon were provisionally identified using the BLASTN algorithm available on NCBI (https://blast.ncbi.nlm.nih.gov/Blast). The similarity indices and query coverage were recorded. A reference sequence library was then constructed for each species which consisted of sequences matching 98%–100% in sequence similarity with 97%–100% query coverage (Larranaga & Hormaza, 2015).

There were significantly different nucleotide lengths sizes for each barcode and the two‐locus combinations prevented acceptable alignment for several species. It was, therefore, difficult to construct multilocus barcodes using the same alignment associated with the corresponding single‐locus barcodes for each taxon in the reference dataset. This difficulty has been reported by others (Hollingsworth et al., 2016). As such, analyses were conducted for separate barcodes.

Sequences were aligned using MAFFT v7 (http://mafft.cbrc.jp/alignment/software/, Katoh, 2013). The aligned sequences were examined visually, and manual adjustments were made to ensure common start and end lengths. MEGA7 software (Tamura et al. 2011) was used to calculate pairwise distances among the aligned sequences using the Kimura 2‐parameter model (Kimura, 1980) to assess intra‐ and interspecies differences.

### 2.5 DNA polymorphism analysis

The level of DNA polymorphism of the aligned sequences of each reference sequence dataset was carried out using DnaSP software (http://www.ub.edu/dnasp/; Rozas, 2009; Librado & Rozas, 2009). DNA polymorphism analysis is an approach that can potentially identify important diagnostic differences among sequences that are not detected by distance or tree‐based query assignment methods (DeSalle, Egan, & Siddall, 2005; Pettengill & Neel, 2010). This approach has not been previously applied for analyzing barcode sequences, and this is the first reported use here.

### 2.6 Direct sequence comparison

The “Species Identifier” suite of tools in the Taxonomy‐aware DNA sequence processing toolkit (TaxonDNA; http://taxondna.sourceforge.net/; Meier, Kwong, Vaidya, & Ng, 2006) was used to explore intra‐ and interspecific genetic distances, matching sequences, and clustering sequences based on pairwise distances. Genetic distance data was used to implement a threshold for determining species identity. This threshold represented the pairwise genetic distance at which 95% of all conspecific individuals were correctly classified. There must be multiple accessions of most species in the reference sequence database, and conspecifics should be present in the database in order to apply the threshold value. BLAST analysis produced similarity hits with sequences belonging to genera outside of the query sequence and as such, demonstrated close genetic affinities to other congeneric species. These sequences were, therefore, also included in the reference sequence dataset.

To evaluate species identity success, the criteria “Best Match” and “Best Close Match” implemented in TaxonDNA were evaluated. “Best Match” is designated if the query sequence is assigned to the genus of the most similar reference sequence. “Best Close Match” is designated if the query sequence is assigned to the genus of the most similar library sequence based on K‐2‐P distance threshold. A query that falls below the determined threshold value will be classified as unidentified (“no match”). The Barcode of Life Data Systems (BOLD), assigns identities using a pairwise genetic distance threshold of 1% for animal species (Ratnasingham & Hebert, 2007). However, the threshold has to be determined for each taxon as it is expected that there is no common threshold value across several different taxonomic groups (Rach, DeSalle, Sarkar, Schierwater, & Hadrys, 2008).

Assignment of each query sequence to a specific taxon was attempted with three possible outcomes for “Best Match” and “Best Close Match” analyses: (i) A “correct” assignment (i.e., the query was assigned to a taxon), (ii) an “ambiguous” assignment (i.e., if there were no barcodes in the library within the set threshold, the assignment was considered to be “ambiguous”), and an “incorrect” assignment (i.e., the query was not assigned to a taxon). A “correct” assignment was then checked with the morphology‐based identification and if there was concordance, the assignment was considered to be TRUE, or FALSE if there was disagreement with the morphology‐based identification (Ross, Murugan, & Li, 2008; Wilson et al., 2011). An “ambiguous” assignment was concluded where the true taxon based on morphological descriptors was not represented in the reference dataset for that barcode which meant that the library was incomplete with inadequate coverage for that specific taxon (Ross et al., 2008; Wilson et al., 2011).

### 2.7 Tree‐based analysis

Neighbor‐joining (NJ; Saitou & Nei, 1987) analysis was carried out to determine phylogenetic placement of a query sequence in relation to a reference sequence dataset. Bootstrap values >70% at a given branch were considered strong support for the existence of that branch. The model implemented was the K‐2‐P genetic distance model with 1,000 pseudoreplicates (Felsenstein 1985). Using tree‐based criteria, query sequences are assigned to a species when they clustered with barcodes from their correct taxon with high bootstrap support (Elias et al., 2007; Mao et al., 2014). Controversy arises when correct assignment via this method of analysis requires that the taxon be monophyletic and when deep phylogenies cannot be tracked. The NJ algorithm is used here to determine clustering of closely related individuals and not as an absolute confirmation of taxon identification.

## 3 RESULTS

### 3.1 PCR and sequencing

Table 3 summarizes the outcome of PCR amplification and sequencing after optimization. With respect to sequencing, the *rpoC1* PCR product was the most difficult to sequence. *rbcL* gave the cleanest reads compared to *rpoC1* and *matK*. These sequences also had zero InDels for all species except *Aristolochia*. The *rbcL* sequences of the reference library mined from GenBank had fewer ambiguous bases (“M,” “S,” “Y,” “K,” “N,” “W”) compared to *matK* and were easily aligned.

**Table 3:** PCR amplification and sequencing success

Species	Primer success	PCR amplification after optimization	Best sequence reads^a^	Worst sequence reads^b^
*Acalypha grisebachiana*	*rbcL*;* rpoC1*	100%	*rbcL*	*rpoC1*
*Aristolochia boosii*	*matK*;* rbcL*;* rpoC1*	100%	*rbcL*	*rpoC1*
*Begonia mariannensis*	none	N/A	N/A	N/A
*Clusia aripoensis*	*rbcL*	100%	*rbcL*	*rpoC1*
*Clusia tocuchensis*	none	N/A	N/A	N/A
*Ilex arimensis*	*matK*;* rbcL*;* rpoC1*	100%	*rbcL*	*rpoC1*
*Macrolobium trinitense*	none	N/A	N/A	N/A
*Maytenus monticola*	*matK*;* rbcL*;* rpoC1*	100%	*rbcL*	*rpoC1*
*Metastelma freemani*	*matK*;* rbcL*;* rpoC1*	100%	*rbcL*	*rpoC1*
*Myrcia stenocarpa*	None	N/A	N/A	N/A
*Philodendron simmondsii*	*matK*;* rbcL*;* rpoC1*	100%	*rbcL*	*rpoC1*
*Scleria orchardii*	None	N/A	N/A	N/A
*Clusia intertexta*	None	N/A	N/A	N/A
*Xyris grisebachii*	None	N/A	N/A	N/A

a Best sequence reads—clear reads without incorporation of ambiguous bases.

b Worst sequence reads—sequence reads with numerous ambiguous bases, base deletion or addition, premature termination of sequence.

### 3.2 DNA polymorphism analysis

**Figure 2:**
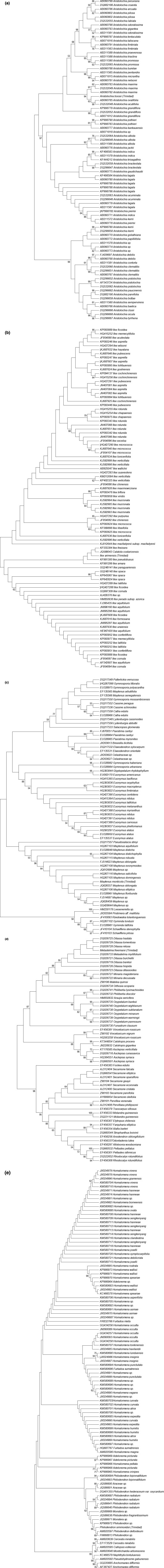
(a–e) Neighbor‐joining tree for five species based on *matK* sequences. Clustering of all query sequences of species under study was inferred using the neighbor‐joining method in MEGA6. The condensed tree (50% bootstrap consensus tree) showing only clustering topology is presented and the percentage of replicate trees in which the associated taxa clustered together in the bootstrap test (1,000 replicates) is indicated next to the branches. The genetic distances were computed using the Kimura 2‐parameter method and are in the units of the number of base substitutions per site. All positions containing gaps and missing data were eliminated. a—*Aristolochia boosi*, b—*Ilex arimensis*, c—*Maytenus monticola*, d—*Metastelma freemani*, e—*Philodendron simmondsii*

**Figure 3:**
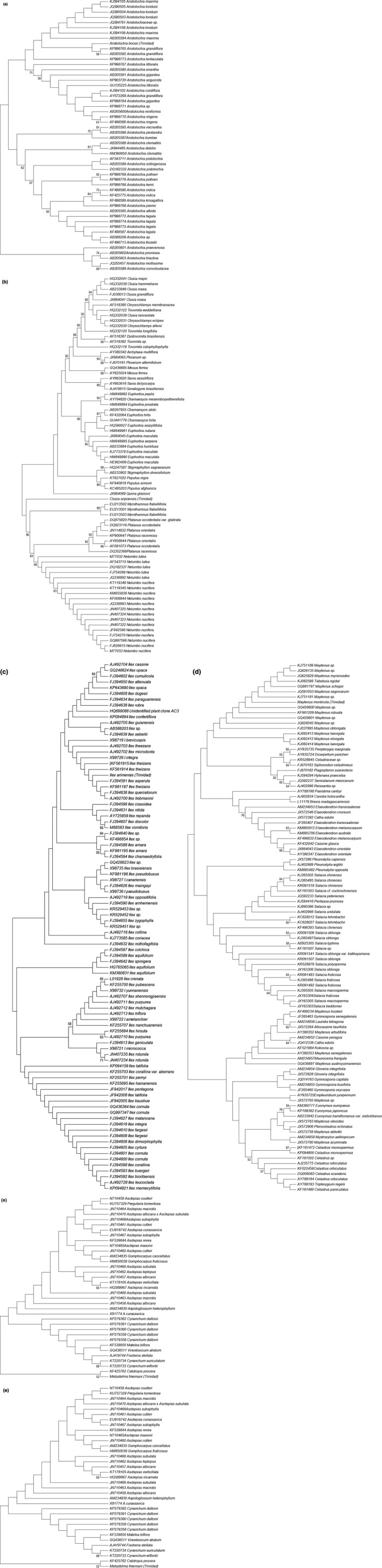
(a–f) Neighbor‐joining tree for six species based on *rbcL* sequences. Clustering of all query sequences of species under study was inferred using the neighbor‐Joining method in MEGA6. The condensed tree (50% boot strap consensus tree) showing only clustering topology is presented, and the percentage of replicate trees in which the associated taxa clustered together in the bootstrap test (1,000 replicates) is indicated next to the branches. The genetic distances were computed using the Kimura 2‐parameter method and are in the units of the number of base substitutions per site. All positions containing gaps and missing data were eliminated. a—*Aristolochia boosi*, b—*Clusia aripoensis*, c—*Ilex arimensis*, d—*Maytenus monticola*, e—*Metastelma freemani*, f—*Philodendron simmondsii*

Tables 4 and 5 summarizes the DNA polymorphism detected in the *matK* and *rbcL* sequences. *matK* sequences had a higher level of polymorphism and had more parsimony informative sites than *rbcL* sequences regardless of species. *matK* sequences also enabled a higher percentage of correct identifications compared to *rbcL* regardless of species. *matK* sequences yielded a higher number of BLASTN hits to the same genus as the query sequence regardless of species. *matK* sequences extracted from GenBank had “R,” “Y,” “N,” “S,” “K,” and “M” bases. *matK* sequences had a higher number of nucleotide differences than *rbcL* sequences. *matK* sequences in the reference library dataset had lower CT and C values compared with *rbcL* sequences especially for *Aristolochia*,* Ilex,* and *Philodendron* species. GenBank accession numbers for all references sequences used in this study are indicated in the NJ trees inferred for each species (Fig. 2a–e for *matK* sequences and Fig. 3a–f for *rbcL* sequences).

**Table 4:** DNA polymorphism data for the *matK* barcode

Marker	DNA Polymorphism Parameters	*Aristolochia boosii*	*Ilex arimensis*	*Maytenus monticola*	*Metastelma freemani*	*Philodendron simmondsii*
*matK*	*N*	81	89	65	57	101
	Aligned sequence length (nt)	815	698	628	799	715
	# monomorphic sites	582	657	640	581	615
	# polymorphic sites	214	41	128	188	82
	# singleton sites	58	21	69	75	31
	# parsimony informative sites	150	20	59	113	50
	# indel sites	24	0	0	30	54
	# mutations (Eta)	55	41	140	223	88
	# nucleotide differences (*k*)	29.206	3.061	11.641	30.031	7.754
	Nucleotide diversity (π)	0.039	0.004	0.015	0.038	0.011
	Conservation threshold (CT)	0.83	1	0.93	0.83	0.98
	Sequence conservation (C)	0.734	0.941	0.833	0.734	0.884
	Conservation *P*‐value	NCRF	Region 1 = 0.022 (nt370–429 Region 2 = 0.004 (nt435–518)	Region = 0.003 (nt655–745)	Region = 0.011 (nt1–83)	NCRF

NCRF, No conserved region found.

**Table 5:** DNA polymorphism data for the *rbcL* barcode

Marker	DNA Polymorphism Parameters	*Aristolochia boosii*	*Ilex arimensis*	*Maytenus monticola*	*Metastelma freemani*	*Philodendron simmondsii*	*Acalypha grisebachiana*	*Clusia aripoensis*
*rbcL*	*N*	52	98	35	101	97	30	71
	Aligned sequence length (nt)	527	516	503	527	521	517	526
	# monomorphic sites	463	492	448	512	460	481	436
	# polymorphic sites	43	24	55	14	61	36	82
	# singleton sites	17	10	28	9	18	18	14
	# parsimony informative sites	26	14	27	5	43	18	68
	# indel sites	21	0	0	0	0	0	8
	# mutations (Eta)	49	26	59	14	74	36	96
	# nucleotide differences (*k*)	6.675	3.146	4.747	1.402	10.189	5.524	8
	Nucleotide diversity (π)	0.013	0.006	0.009	0.003	0.019	0.011	0.043
	Conservation threshold (CT)	1	1	0.99	1	0.98	1	0.94
	Sequence conservation (C)	0.915	0.953	0.891	0.973	0.893	0.93	0.844
	Conservation *P*‐value	Region 1 = 0.029 (nt32–69)	Region 1 = 0.006 (nt28–125)	NCRF	Region = 0.016 (nt53–186)	NCRF	Region 1 = 0.039 (nt30–72)	Region = 0.002 (nt13–92)
		Region 2 = 0.048 (nt72–104)	Region 2 = 0.041 (nt372–434)				Region 2 = 0.036 (nt74–117)	
		Region 3 = 0.011 (nt151–198)					Region 3 = 0.028 (nt239–285)	
		Region 4 = 0.039 (nt274–308)					Region 4 = 0.028 (nt455–501)	
		Region 5 = 0.039 (nt403–437)						

NCRF, No conserved region found.

BLASTN resulted in mixed hits for *rbcL* sequences for 50% of the species under study. However, *Aristolochia* and *Ilex* sequences resulted in 100% genus hits for both *matK* and *rbcL* sequences. *rbcL* sequences extracted from GenBank were clean reads with few sequences having “R,” “Y,” “N,” “S,” “K,” and “M” bases. *rbcL* sequences of the endemic species also had a higher query coverage (99%–100%) and higher similarity (99%) compared with *matK* sequences. *rbcL* sequences also had fewer InDels than *matK* sequences and contained several regions or blocks of nucleotides with conserved sequences which resulted in high CT (CT = 1) and C values (close to 1).

### 3.3 Sequence identification

Specimen identification success for the two markers, *matK* and *rbcL,* is outlined in Tables 6 and 7. There was a higher proportion of correctly identified species obtained with *matK* sequences compared with *rbcL* sequences. *rbcL* sequences had a higher proportion of ambiguously classified sequences compared to *matK* sequences. The *matK* reference dataset also had a higher number of conspecifics compared with the *rbcL* reference dataset. In terms of genetic distance, *Aristolochia* sequences shared the highest distance and *Metastelma* sequences shared the lowest distance, regardless of marker, when compared with other species according to K‐2‐P analysis. All of the endemic species from Trinidad, except *Clusia aripoensis,* shared a common classification as “ambiguous” regardless of marker. *Clusia aripoensis* could not be matched according to TaxonDNA's “best match” and “best close match” criteria, and there was no placement of this species into genus‐specific cluster in the NJ tree generated by the K‐2‐P model.

**Table 6:** Kimura 2‐parameter threshold data and sequence matches in the reference library

Marker	Species	K‐2‐P pairwise distance and threshold (%)	Sequences with at least one matching sequence in the dataset	Sequences with at least one matching conspecific sequence in the dataset	Sequences with a closest match at 0%
*matK*	*Aristolochia boosii*	4.96	80	48	29
	*Maytenus monticola*	2.92	63	18	21
	*Metastelma freemanii*	0.25	56	8	11
	*Philodendron simmondsii*	0.53	100	76	76
	*Ilex arimensis*	0.72	84	63	68
*rbcL*	*Aristolochia boosii*	1.72	52	30	32
	*Maytenus monticola*	0.79	101	43	65
	*Metastelma freemanii*	0.19	34	16	28
	*Philodendron simmondsii*	0.57	97	32	55
	*Ilex arimensis*	1.16	83	31	59
	*Acalypha grisebachiana*	1.75	30	20	19
	*Clusia aripoensis*	0.19	71	37	58

**Table 7:** Species identification success based on best match and best close match analyses

Marker	Species	Best match criterion			Best close match criterion			Trinidad species ID classification
		Correct identification (%)	Ambiguous Identification (%)	Incorrect Identification (%)	Correct identification (%)	Ambiguous Identification (%)	Incorrect Identification (%)	
*matK*	*Aristolochia boosii*	34 (42.5)	13 (16.25)	33 (41.25)	34 (42.5)	13 (16.25)	33 (41.25)	Ambiguous
	*Maytenus monticola*	12 (18.75)	24 (37.5)	28 (43.75)	12 (18.75)	24 (37.5)	28 (43.75)	Ambiguous
	*Metastelma freemanii*	8 (14.28)	13 (23.21)	35 (62.5)	8 (14.28)	13 (23.21)	35 (62.5)	Ambiguous
	*Philodendron simmondsii*	17 (17.0)	66 (66.0)	17 (17.0)	17 (17.0)	66 (66.0)	13 (13.0)	Ambiguous
	*Ilex arimensis*	16 (19.04)	58 (69.04)	10 (11.90)	16 (19.04)	58 (69.04)	10 (11.90)	Ambiguous
*rbcL*	*Aristolochia boosii*	11 (21.15)	28 (53.84)	13 (25.0)	11 (21.15)	28 (53.84)	13 (25.0)	Ambiguous
	*Maytenus monticola*	13 (12.87)	60 (59.4)	28 (27.72)	13 (12.87)	59 (58.41)	27 (26.73)	Ambiguous
	*Metastelma freemanii*	0 (0)	29 (85.29)	5 (14.7)	0 (0)	24 (70.58)	4 (11.76)	Ambiguous
	*Philodendron simmondsii*	6 (6.18)	56 (57.73)	35 (36.08)	6 (6.18)	50 (51.54)	33 (34.02)	Ambiguous
	*Ilex arimensis*	3 (3.61)	68 (81.92)	12 (14.45)	3 (3.61)	68 (81.92)	12 (14.45)	Ambiguous
	*Acalypha grisebachiana*	4 (13.33)	19 (63.33)	7 (23.33)	4 (13.33)	19 (63.33)	7 (23.33)	Ambiguous
	*Clusia aripoensis*	24 (33.8)	36 (50.7)	11 (15.49)	24 (33.8)	28 (39.43)	6 (8.45)	No match

### 3.4 Reference dataset coverage

Library representation and similarity in the BLAST reference library for each species and marker are summarized in Table 8 and 9. Coverage was representative for only *Aristolochia*,* Ilex,* and *Philodendron matK* sequences. Reference dataset coverage was representative for *Aristolochia*,* Ilex*, and *Acalypha rbcL* sequences. A minimum cutoff value for query coverage was applied at 97% if the subject sequence belonged to the same genus as the query sequence. Similarly, a minimum similarity value was applied at 97% if the subject sequence belonged to the same genus as the query sequence with the exception of *Clusia aripoensis* which only had 94% maximum similarity regardless of genus. BLASTN hits for *Clusia aripoensis* also revealed poor reference dataset coverage with the lowest similarity scores (94%) of all the species included in this study. The distances among the conspecifics in the dataset were also very low. This may explain the “no match” designation for *Clusia aripoensis*.

**Table 8:** Clustering analysis of *matK* sequences based on K‐2‐P genetic distances and the neighbor‐joining algorithm

Marker	Species	Reference dataset sequence coverage	K‐2‐P pairwise distance and threshold (%)	Query coverage %; Similarity %	Placement of Trinidad sequence	Bootstrap score (bs) for Trinidad placement	Overall cluster support	Polytomies present (Yes/No)
*matK*	*Aristolochia boosii*	Representative (100% belonged to Aristolochia genus)	4.96	99%–100%; 96%–98%	Clustered with A. maxima and A. ovalifolia	94%	Majority >90%	No
	*Ilex arimensis*	Representative (100% belonged to Ilex genus)	0.72	94%–99%; 99%		<50%	Majority <50%; but 12 clusters with bs >60%	Yes
	*Maytenus monticola*	50% of sequences belonged to Maytenus or to Euonymus genus which are synonyms	2.92	98%–100%; 99%	Clustered with only Maytenus sequences	96%	Maytenus cluster 96%; Euonymus cluster 88%; All other sequences clustered according to genus with high bs support (>75%)	No
	*Metastelma freemani*	Not representative (14% of sequences belonged to either Metastelma or Ditassa genus which are synonyms)	0.25	99%; 96%–99%	Clustered with all other Metastelma and Ditassa sequences	86%	All other sequences clustered according to genus with high bs support (>75%)	No
	*Philodendron simmondsii*	Representative (90% of sequences belonged to either Philodendron or Homalomena genus which are synonyms)	0.53	100%; 99%–100%	Clustered with P. radiatum sequences	62%	Majority of clusters had low bs support (bs < 50%) but five clusters had bs >60%	Yes

**Table 9:** Clustering analysis of *rbcL* sequences based on K‐2‐P genetic distances and the neighbor‐joining algorithm

Marker	Species	Reference dataset sequence coverage	K‐2‐P pairwise distance and threshold (%)	Query coverage %; Similarity %	Placement of Trinidad sequence	Bootstrap score	Overall cluster support	Polytomies present (Yes/No)
*rbcL*	*Aristolochia boosii*	Representative (100% of sequences belonged to Aristolochia genus)	1.72	97%–99%; 97%–99%	Clustered with A. maxima and A. tonduzu with low bs support	<50%	Most other clusters were moderately supported (bs >60%)	Yes
	*Ilex arimensis*	Representative (100% of sequences belonged to Ilex genus)	1.16	99%; 99%–100%	Clustered with low bs support	<50%	Majority of clusters had low bs support (<50%); only four clusters had bs >60%	Yes
	*Maytenus monticola*	Not representative (14% of sequences identified as belonging to Maytenus genus)	0.79	98%–100%; 99%	Clustered with only Maytenus sequences but with low bs support	<50%	Majority of clusters had low bs support (<50%)	Yes
	*Metastelma freemani*	Not representative (29% of sequences identified as belonging to either Metastelma or Cynanchum genus)	0.19	98%–100%; 99%	Trinidad sequence positioned in a separate cluster from all other sequences	<50%	Majority of clusters had low bs support (<50%)	Yes
	*Philodendron simmondsii*	Not representative (13% of sequences belonged to either Philodendron or Homalomena genus	0.57	99%; 98%–100%	Clustered with other Philodendron and Homalomena sequences with high bs support	73%	Majority of main clusters had moderate bs support (>60%)	Yes
	*Acalypha grisebachiana*	Representative (100% of sequences belonged to Acalypha genus)	1.75	97%–100%; 99%–100%	Clustered with one of two main clusters, the first consisted of the majority of sequences but with no calculated bs score; the second of the two clusters was highly supported (99%)	Not available	Main clusters had moderate bs support (>60%); majority of clusters however, had low bs support (>50%)	Yes
	*Clusia aripoensis*	Not representative (8.5% of sequences belonged to Clusia genus)	0.19	100%; 94%	Did not cluster with any other species including those 6 belonging to Clusia genus	Not available	Main clusters had moderate to high bs support (>60%–90%); clusters were mostly genus‐specific	Yes

BLAST searches revealed very poor reference dataset coverage for all species for the *rpoC1* marker. *Aristolochia* had only 11 sequences belonging to this genus (97%–100% query coverage; 97%–98% similarity); *Acalypha* had no other sequences belonging to this genus; *Ilex* had four sequences belonging to this genus (96%–100% query coverage; 98% similarity); *Maytenus* had 15 (98%–100% query coverage; 99% similarity); *Metastelma* had just five other sequences belonging to this genus (100% query coverage; 97%–98% similarity); *Philodendron* had no other sequences belonging to this genus. In most cases, the BLAST reference dataset also included other sequences belonging to at least 15 other genera designated as hits with the same query coverage and similarity as those sequences belonging to the same genus as the query sequence. Further analysis was, therefore, not carried out for this marker.

### 3.5 Clustering of query sequences in NJ trees

*matK* sequences allowed specific placement of Trinidad species within genus‐specific clusters with moderate to high bootstrap support (>60%–90%; Table 8; Fig. 2a–e), for all but one species, *Ilex arimensis,* which was not placed into a discernible cluster even though the reference dataset was representative (100%), query coverage and similarity of sequences in this reference dataset was optimal (94%–99%; 99%), but whose sequence variation was low for this marker (0.72%). Polytomies were also evident in the NJ tree generated for *Ilex* and *Philodendron*.

*rbcL* sequences did not allow specific placement of Trinidad species into genus‐specific clusters with moderate to high bootstrap support (>60%) except for *Philodendron simmondsii* (bs = 73%; Table 9; Fig. 3a–f). Although sequences generally were positioned in genus‐specific clusters, these were not well supported (bs < 50%). There were also polytomies in the NJ trees constructed for *rbcL* sequences for all species. K‐2‐P genetic distances were lower for *rbcL* sequences than for *matK* sequences.

## 4 DISCUSSION

In this study, we examined the suitability of the proposed CBOL Plant Working Group barcoding markers for land plants to confirm the identity of specimens of 14 endemic and rare vascular plant species in Trinidad. Our results indicated that 50% of the species under study were not identified using a barcoding approach due to amplification failure. It was evident that the quality of DNA was an important factor in amplification success and PCR failure may be a result of DNA quality and not necessarily poor primer annealing. The method of DNA extraction and quality of DNA are critical to successful amplification. Others explained amplification failure as a result of poor annealing with standard *matK* or *rbcL* primers and highlighted the need to redesign species‐specific primers (Kress & Erickson, 2007; Sass et al. 2007; Fazekas et al., 2008; Lahaye et al., 2008; Casiraghi et al., 2010; Roy et al., 2010). According to Casiraghi et al. (2010), *matK* sequences were analyzed in different plants but the universality of this barcode ranged from routine success to low recovery. Casiraghi et al. (2010) also acknowledged that even the most conserved *rpoB*,* rpoC1,* and *rbcL* or a portion of *matK* that demonstrates a rapid rate of evolution, in some plant families, these genes are difficult to amplify. For example, *matK* and *rbcL* were able to identify species to the *Betus* and *Salix* genus level, but did not allow adequate resolution to distinguish among species belonging to these genera and the rate of amplification was low (only 21% of the *Salix* samples amplified; Jarvinen et al. 2004; Fazekas et al., 2008; von Crautlein et al., 2011).

DNA barcoding can be suitable for two different purposes: (i) the molecular identification of already described species, and (ii) the discovery of undescribed species (Casiraghi et al., 2010). In a typical DNA barcoding strategy, the sequence of a given species is compared against reference sequences in a library database (sequences of previously identified individuals) for a given barcode. This comparison can result in a query sequence match to another sequence in the library, which leads to species identification (Hajibabaei, Singer, Hebert, & Hickey, 2007). A case where there is no match to any record in the database could also indicate the existence of a new species (Hebert et al. 2004). Trinidad sequences were accurately identified to the genus level for all endemic plant species successfully amplified and sequenced using both *matK* and *rbcL* markers. Accurate genus–level identification is important for poorly described (or sampled) groups as well as for the enforcement of quarantine and trafficking regulations as regulators more commonly list genera rather than species (Little, 2011). In this study, our endemics did not match any other species with 100% similarity in the reference libraries created for each Trinidad species. Does this mean new species assignments for Trinidad endemics? Casiraghi et al. (2010) cautions against assigning biological meaning to genetic ranks, unless these sequences are able to clearly and unequivocally link a species to the variability pattern of a single DNA barcoding marker.

There is no single optimal method to determine the resolving power of DNA barcodes for all taxa (Austerlitz et al., 2009; Casiraghi et al., 2010; Collins & Cruickshank, 2013; Meyer & Paulay, 2005; Moritz & Cicero, 2004; Ross et al., 2008). Different approaches exist for matching an unknown query sequence with sequences in a reference database or library and tend to be based on ad hoc criteria which may include the frequency of the highest hits, percentage sequence similarity, bootstrapping, BLAST scores or tree‐based clustering assessment (Kress et al., 2009; Wilson et al. 2011). Although there is no consensus on the “best approach” and in reality, the most appropriate approach may be dependent on a number of variables, it is recommended that, as far as possible, the taxonomic origin and assignments be independently confirmed (i.e., using morphological characters) to improve the accuracy of taxonomic assignment through barcoding (Hollingsworth et al., 2016; Wilson et al. 2011).

The main challenge to using distance‐based methods to species identification is that no single genetic distance threshold distinguishes all species (Ferguson, 2002; DeSalle et al., 2005; Little and Stevenson 2007; Wilson et al. 2011). A threshold value calculated from genetic distances may be more appropriate than using a single arbitrary 1% or 3% threshold (Meier et al., 2006; Fazekas et al. 2009; Collins & Cruickshank, 2013). In this study, there was little change in the proportion of “correct,” “ambiguous,” and “incorrect” assignments when threshold values of 1%, 3% and a separate calculated threshold for each reference sequence library dataset were used. Despite using threshold values calculated from K‐2‐P genetic distances for each taxon, all of the endemic species were still classified as “ambiguous” but, they were all assigned to the correct genus for *matK* and *rbcL* barcodes. *Clusia aripoensis* was the only species with a “no match” status based on *rbcL* sequence comparisons. Two reasons for this result may be explained as: (i) there was poor library sequence database coverage, and (ii) genetic distances were higher than the calculated threshold for this taxon. In this study, DNA barcoding was useful in flagging atypical specimens or in identifying cryptic species for further taxonomic investigation (Hajibabaei et al., 2007).

The low rate of “correct” classification for both methods that provide “ambiguous” and “no match” classifications are important because they reveal several gaps in the approach to analysis including (i) reference sequence library coverage, (ii) low genetic variation among barcode sequences, and (iii) whether markers are targeting regions of the genomes whose genetic distances can vary from species to species (Hollingsworth et al., 2016). Therefore, the need for further research into understanding the cause of the “ambiguous” or “no match” status in identity is highlighted. One approach to ensure good reference library coverage would be to barcode congenerics for each species selected for study sharing the same geography. Even if this were feasible, in terms of availability of specimens, there is no guarantee that these congeneric barcodes would be sufficient to discriminate among all species as was found to be the case with Dendrobium species (Singh, Parveen, Raghuvanshi, & Babbar, 2012).

Tree‐based methods involve assignment of a query sequence to a certain taxon if it is found in a clade consisting of reference sequences with moderate to high bootstrap support. These methods require appropriate alignment of all sequences which may be difficult for highly divergent sequences (Mao et al., 2014; Wilson et al. 2011). While barcode libraries are somewhat similar to molecular phylogenetic data (i.e., they are both built from sequence information from different species), DNA barcodes do not usually have sufficient phylogenetic signal to infer evolutionary relationships (Hajibabaei et al., 2006, 2007). In this study, NJ trees were used to establish clustering of query sequences into correct genus‐specific groups with strong bootstrap support and were not used to infer phylogeny. Poor resolution in tree topologies with low bootstrap scores and polytomies obtained for *rbcL* sequences were obtained which may be due to inadequate low genetic distances for most species (Hebert et al., 2003; Hollingsworth et al., 2016; Kress et al., 2009; Ross et al., 2008; Wilson et al. 2011). Others have reported low resolution in *rbcL* because it is known to have insufficient nucleotide sequence variability to distinguish among closely related species (Kress & Erickson, 2007; Newmaster, Grguric, Shanmughanandhan, Ramalingam, & Ragupathy, 2013).

In this study, it was difficult to concatenate relevant sequences mined from GenBank for the *matK* and *rbcL* markers for each species. As such, we analyzed separate markers. Hollingsworth et al. (2016) also reported on the difficulty in concatenating sequences available in reference libraries. Others found no improvement in species identification using a combined multilocus approach and loci rarely discriminated among samples that were not already correctly classified using the better performing of the two loci separately (Lahaye et al., 2008). In fact, it seems counterintuitive to combine a high‐performing marker with a low performing marker in an effort to improve the proportion of correct assignments. In this study, the *matK* marker had a higher percentage of correct identifications compared to the *rbcL* marker.

## 5 CONCLUSIONS

DNA barcoding has the potential to distinguish among species that are closely related and among those which are evolutionarily divergent using single barcodes. We have found that barcoding success is dependent on having taxonomically appropriate representation in the reference sequence database, the genetic distance among the sequences in this database, the species under study which affects both technical and species discrimination success, the accuracy of identity of species in the reference sequence database, the barcodes used and whether there is a high level of monophyly among species of a given genus. In other words, the performance of the *matK* and *rbcL* approved barcodes appeared to be species‐specific or genus‐specific, which is what has been cautioned by others (Casiraghi et al., 2010). The “best close match” tool implemented in the TaxonDNA suite was useful because of its ability to discriminate among “correct,” “ambiguous,” “incorrect,” and “no match” classifications for each species in the reference sequence database in addition to query sequences. The tree‐based method generally reflected the genetic distances among the sequences in the reference sequence database, and in most cases, our endemic species were positioned in clusters that were genus‐specific based on the *matK* barcode. This was not the case for the *rbcL* barcode as the tree topology was poorly resolved due to very low variation among the sequences of the reference sequence database. Others have used different barcodes such as *ITS2* in similar ethno‐pharmacology‐based identifications with success (Chen et al., 2010; Gao et al., 2010). DNA barcoding also involves massive sample sets with often industrial‐scale laboratory practices and bioinformatics pipelines (Hollingsworth et al., 2016). These challenges are especially important to developing countries with high levels of biodiversity but with limited resources to conduct DNA barcoding work.

## CONFLICT OF INTEREST

The authors declare that they have no conflict of interest.
